# Predictors of therapeutic alliance in two treatments for adults with severe and enduring anorexia nervosa

**DOI:** 10.1186/s40337-016-0102-6

**Published:** 2016-04-05

**Authors:** Colleen Stiles-Shields, Bryony H. Bamford, Stephen Touyz, Daniel Le Grange, Phillipa Hay, Hubert Lacey

**Affiliations:** Department of Preventive Medicine, Northwestern University Feinberg School of Medicine, Chicago, IL USA; The London Centre for Eating Disorders and Body Image, London, UK; University of Sydney, School of Psychology, Sydney, Australia; Department of Psychiatry, University of California, San Francisco, San Francisco, CA USA; Department of Pediatrics, University of California, San Francisco, San Francisco, CA USA; Centre for Health Research, School of Medicine, Western Sydney University, Sydney, Australia; St. George’s, University of London, Eating Disorders Research Group, London, UK

**Keywords:** Therapeutic alliance, Anorexia nervosa, Cognitive behavioral therapy, Specialist supportive clinical management, Predictors

## Abstract

**Background:**

Therapeutic alliance (TA) has been found to be a significant predictor of outcome for patients with severe and enduring anorexia nervosa (SE-AN), accounting for more variance than treatment type. To better understand how to promote TA for this population, the aim of the current study was to investigate predictors of TA in adults with SE-AN.

**Methods:**

Participants were 63 adult females with SE-AN presenting to an outpatient, multi-site randomized controlled trial conducted at two clinical sites. Participants’ perception of the quality of their therapeutic relationship, demographic information, and eating disorder symptomatology were assessed via interview and questionnaire measures.

**Results:**

Baseline ratings of how successful participants believed treatment would be for them was the only variable to significantly predict early (*p* = .01), mid (*p* = .009), and late treatment alliance (*p* = .03). No other variables investigated predicted the quality of patient rated TA at any point in treatment (*ps* > .57).

**Conclusions:**

Results suggest instilling hope in treatment outcome may enhance TA, and in turn, outcomes for patients with SE-AN in outpatient therapy.

## Background

Therapeutic alliance (TA) is a relational bond that develops between a patient and clinician via collaborative work and trust, as both endeavor to establish and accomplish treatment goals [1]. Cited as a critical non-specific factor in the treatment of depression [2], TA has been shown to predict outcomes across a variety of treatments and disorders, including in chronic presentations [3–10]. In the treatment of eating disorders, TA is often considered an important element of interventions, but with mixed effect on treatment success [11–16].

TA has been shown to play a central role in preventing premature treatment drop-out in an inpatient population with anorexia nervosa (AN) [17, 18], with even initial impressions promoting treatment compliance [17]. Further, in a sample of adults with severe and enduring anorexia nervosa (SE-AN), TA was found to be a significant predictor of eating disorder symptoms at end of treatment (EOT) and at follow-up [19]. Indeed, TA actually accounted for more of the variance in outcome than treatment type. As such, correlates of TA are a critical domain to explore for patients with SE-AN.

While TA is recognized as an important predictor of treatment engagement, less is known about the factors that may contribute to the development of a strong alliance in patients with AN, particularly those with a severe and enduring course of illness. Research within the field of anxiety and depression indicates that patients with more severe symptoms report poorer TA [20]; however, these associations between symptom severity and TA have not been replicated in adults with bulimia nervosa (BN) [21]. The quality of the therapeutic relationship has been noted as worse in individuals with chronic eating disorders [22], suggesting that duration of illness, or number of previous treatment episodes may adversely affect treatment engagement and TA. Additionally, when exploring psychotherapy processes, patient preconceptions and expectations regarding improvement have been found to be associated with stronger alliance and better overall treatment outcomes [23, 24]. This finding has been supported within a treatment trial for BN [21]. Further, there is recent evidence to suggest that positive early symptom change may be responsible for enhancing TA in patients with BN [11, 14]. Despite these findings, there is still a dearth of research investigating whether, and which, patient characteristics influence TA in SE-AN. Greater understanding of patient characteristics that may influence therapeutic alliance and treatment engagement is needed to help clinicians identify specific treatment adaptations that may be needed to better tailor treatment to individual need.

The aim of this paper is to explore possible correlates of TA that may be present, irrespective of treatment or therapist variation. In line with previous research highlighted above, we hypothesized that characteristics associated with SE-AN (i.e., longer treatment duration, increased severity, higher previous treatment episodes, lower motivation and expectation of treatment success, and lower body mass index) would predict poorer participant-rated TA for adults with SE-AN.

## Methods

This study is a secondary analysis of data from a randomized clinical trial (RCT) conducted at two clinical sites (The University of Sydney and St. George’s Hospital, University of London), with a Data and Coordinating Center (The University of Chicago Medicine). The RCT compared the efficacy of Cognitive Behavioral Therapy for anorexia nervosa (CBT-AN) and Specialist Supportive Clinical Management (SSCM) for a sample of adults with SE-AN. Specific treatment effects have been analysed and are reported in the main outcome paper [25]. Participants received 30 individual, outpatient treatment sessions provided over eight months.

### Participants

Recruitment of participants occurred from July 2007 to November 2010 through advertising to eating disorder clinics, clinicians, and generic websites.

Participants were eligible for randomization if they met the *Diagnostic and Statistical Manual for Mental Disorders*, 4^th^ Edition, Text Revision (DSM-IV-TR) [26] criteria for AN, excluding criterion D (amenorrhea); had an illness duration of at least 7 years; were at least 18 years of age; and were female. Exclusion criteria included presenting with a current manic episode or psychosis; current alcohol or substance abuse or dependence; significant current medical or neurological illness (including seizure disorder), with the exception of nutrition-related alterations that are impacted by weight; current engagement in psychotherapy and being unwilling to suspend such treatment for the duration of their participation in the study; and plans to move beyond commuting distance for the study site in the following 12 months or not living within commuting distance to the study site.

In compliance with the Institutional Review Boards (IRB) of the two intervention sites and the data and coordinating center, participants completed written informed consent prior to assessment. In addition to IRB approval, the trial was approved by the Human Research Ethics Committee (approval number 02-2007/9669) and was registered with the Australian New Zealand Clinical Trials Registry (ACTRN12607000440426).

### Treatments and therapists

Participants were randomized to either CBT-AN or SSCM by a biostatistician independent from both intervention sites. Randomization was conducted using Ephron’s biased coin approach, stratified within sites by: 1) subtype of illness (Restrictive and Binge-Purge) and 2) psychopharmacological medication status. Treatment occurred in outpatient settings at The University of Sydney and St. George’s Hospital, University of London. Treatments and therapist characteristics are described in detail in previous papers [25, 27].

### Measures

#### Physical assessment

Participants were weighed in light, indoor clothing with their shoes removed. Weight and height were measured by a trained research assistant using a calibrated digital or balance-beam scale and stadiometer, respectively, to calculate body mass index (BMI = kg/m^2^).

#### Helping Relationships Questionnaire (HRQ)

The HRQ measures the patient’s perspective of the therapist-patient relationship via an 11 self-report items [28]. Items for the HRQ are rated on a 6-point likert scale, ranging from -3 (“Strongly feel it is not true”) to +3 (“Strongly feel it is true”). Total scores, computed by summing all items, range from -33 to 33, with higher total scores reflecting greater TA. The HRQ was administered at week two, mid-treatment, and EOT. Participant responses with missing items on the HRQ were excluded from analyses involving HRQ total scores. The HRQ has strong psychometrics and has been shown to correlate with treatment outcome [29, 30].

#### The Eating Disorder Examination (EDE)

The EDE is a semi-structured investigator-based interview measuring cognitive and behavioral symptoms related to eating disorders [31]. The EDE was used to generate DSM-IV-TR diagnoses for an ED and to assess the severity of symptomatology. Subscales include: Weight Concern, Shape Concern, Eating Concern, and Restraint; global scores reflect the overall severity of ED symptoms.

#### The Anorexia Nervosa Stages of Change Questionnaire (ANSOCQ)

The ANSOCQ is a 20-item self-report questionnaire assessing a patient’s readiness for recovery from AN, with higher total scores reflecting greater readiness for recovery [32].

#### The Treatment Suitability and Patient Expectations (TSPE)

The TSPE is a 2-item self-report questionnaire designed to assess a patient’s belief regarding her expectation of improvement in her treatment assignment (“How successful do you think your treatment here will be?”) and suitability of her assigned treatment. The TSPE items are answered using an 11-point scale ranging from 0 (not at all) to 10 (completely acceptable or extremely suitable). The TSPE is given following the first therapy session, once the patient has met with her therapist and is aware of which treatment to which she has been randomized. The patient is asked to comment on “overall” improvement, rather than improvement on any specific domains (e.g., weight gain, quality of life). The TSPE has been utilized in multiple trials with patients with eating disorders [25, 33–35]. Cronbach’s alphas for the TSPE ranged from .75 to .92 over the assessment time points in this trial.

#### Assessment time points

The physical assessment, EDE and ANSOCQ were administered at baseline (pre randomization), session 15, and EOT. The HRQ and the TSPE were given following the first therapy session, session 15, and EOT.

### Data analysis

Stepwise multiple regressions were conducted to investigate the predictive utility of patient motivation, believed suitability of treatment, symptom severity, duration of illness, number of previous specialist ED treatment experiences, and BMI on early treatment alliance. Stepwise multiple regressions were also conducted to investigate the predictive power of the variables detailed above, as well as early treatment alliance, on participant ratings of mid and end of treatment alliance. An alpha level of .05 was used to provide maximum power to identify potential predictors of therapeutic alliance.

Post hoc analyses were conducted to better understand the role of significant predictor(s) of TA. An independent *t*-Test was used to examine any differences occurring based on treatment assignment and a pearson correlation was run to understand the predictor(s) relationship to outcome. To determine if a significant meditational effect was present among the significant predictor(s) of TA, TA, and eating disorder symptom outcomes (defined by EDE global score), the methodology outlined by Baron and Kenny was utilized to evaluate if mediation was supported [36].

## Results

A total of 63 participants were randomized to CBT-AN (*n* = 31) or SSCM (*n* = 32). The range of age for study participants was 20-62 (*M* = 33.4 ± 9.6), with duration of illness ranging from 7 to 49 years (*M* = 16.6 ± 8.5). The mean BMI for the sample was 16.2 (SD = 1.3, range = 11.8-18.5). The majority of participants met criteria for AN restricting subtype (*n* = 47, 74.6 %). No significant differences on any baseline characteristics were found between treatment groups, sites, or group-by-site interactions [25]. Table 1 shows baseline demographics for all predictor variables.

**Table 1:** Baseline characteristics of predictor variables for therapeutic alliance

Variable	Mean	Standard Deviation	Range (Minimum, Maximum)
Age	33.41	9.57	42 (20, 62)
Duration of Illness	16.57	8.45	42 (7, 49)
ANSOCQ	16.57	17.89	77 (0, 77)
BMI	16.20	1.34	6.67 (11.80, 18.47)
EDE Global Score	3.10	1.31	5.26 (.41, 5.67)
Frequency of Specialist ED Interventions	6.80	4.16	16 (2, 18)
TSPE: Suitability of Treatment	7.41	1.78	9 (1, 10)
TSPE: Success of Treatment	7.08	1.75	9 (1, 10)

*Note*. ANSOCQ = The Anorexia Nervosa Stages of Change Questionnaire; BMI = Body Mass Index; EDE = Eating Disorders Examination; ED = Eating Disorders; TSPE = Treatment Suitability and Patient Expectations

Baseline ratings of how successful participants believed treatment would be was the only variable to significantly predict early (*β* = 0.67, SE = 0.94, *p* = .01), mid (*β* = 0.80, SE = 0.80, *p* = .009), and late treatment alliance (*β* = 0.66, SE = 1.98, *p* = .03; see Table 2). Motivation, symptom severity, duration of illness, age, number of previous specialist ED treatment experiences, early therapeutic alliance, and BMI did not significantly predict alliance at any point in treatment (*ps* > .57; see Table 3).

**Table 2:** Significant predictors of therapeutic alliance determined through stepwise regression

Stepwise regression variable	∆R^2^	∆*F*	B	SE	*Β*	*p*
Early Treatment Alliance Model						
Baseline Rating of Anticipated Treatment Success	.45	8.97	2.83	0.94	.67	.01
Mid Treatment Alliance Model						
Baseline Rating of Anticipated Treatment Success	.64	12.68	2.86	0.80	.80	.009
Late Treatment Alliance Model						
Baseline Rating of Anticipated Treatment Success	.44	7.09	5.27	1.98	.66	.03

*Note*. Variables excluded for early treatment alliance model: number of previous specialist eating disorder interventions, baseline motivation score, duration of illness, age, baseline Body Mass Index (BMI), rating of treatment suitability, and baseline eating disorder severity (*ps* > .68); variables excluded for mid and end of treatment alliance models: early treatment rating of therapeutic alliance, number of previous specialist eating disorder interventions, baseline motivation score, duration of illness, age, baseline BMI, rating of treatment suitability, and baseline eating disorder severity (*ps* > .57)

**Table 3:** Variables excluded as significant predictors of therapeutic alliance determined through stepwise regression

Stepwise regression variable	Beta In	*t*	*p*
Early Treatment Alliance Model			
Previous specialist ED intervention	.362	1.73	.12
Baseline ANSOQC Total	.142	.57	.58
Duration of illness	-.052	-.22	.83
Age	-.026	-.11	.92
Baseline BMI	-.173	-.72	.49
Baseline rating of treatment suitability	.278	1.03	.33
Baseline EDE Global score	-.039	-.17	.87
Mid Treatment Alliance Model			
Previous specialist ED intervention	.119	.49	.64
Baseline ANSOQC Total	.366	1.64	.15
Duration of illness	-.353	-1.70	.14
Age	-.435	-1.81	.12
Baseline BMI	-.403	-1.63	.16
Baseline rating of treatment suitability	-.225	-.74	.49
Baseline EDE Global score	-.281	-1.26	.25
Early Treatment HRQ Total	.087	.27	.80
Late Treatment Alliance Model			
Previous specialist ED intervention	-.091	-.34	.74
Baseline ANSOQC Total	-.176	-.61	.56
Duration of illness	-.229	-.86	.41
Age	-.021	-.07	.95
Baseline BMI	.300	.98	.36
Baseline rating of treatment suitability	-.147	-.44	.67
Baseline EDE Global score	-.049	-.18	.86
Early Treatment HRQ Total	.205	.66	.53

Note. ED = Eating Disorder; ANSOQC = The Anorexia Nervosa Stages of Change Questionnaire; BMI = Body Mass Index; EDE = Eating Disorders Examination; HRQ = Helping Relationships Questionnaire

### Patient expectations of treatment success

Participant ratings of anticipated treatment success did not significantly differ based on treatment assignment (CBT-AN *M* = 7.04 ± 1.97, SSCM *M* = 7.17 ± 1.56, *p* = .78). The TSPE item was significantly correlated with EOT (*r*(47) = -.40, *p* = .005) and follow-up (*r*(44) = -.48, *p* = .001) EDE global scores.

### Mediation analyses

**Fig. 1:**
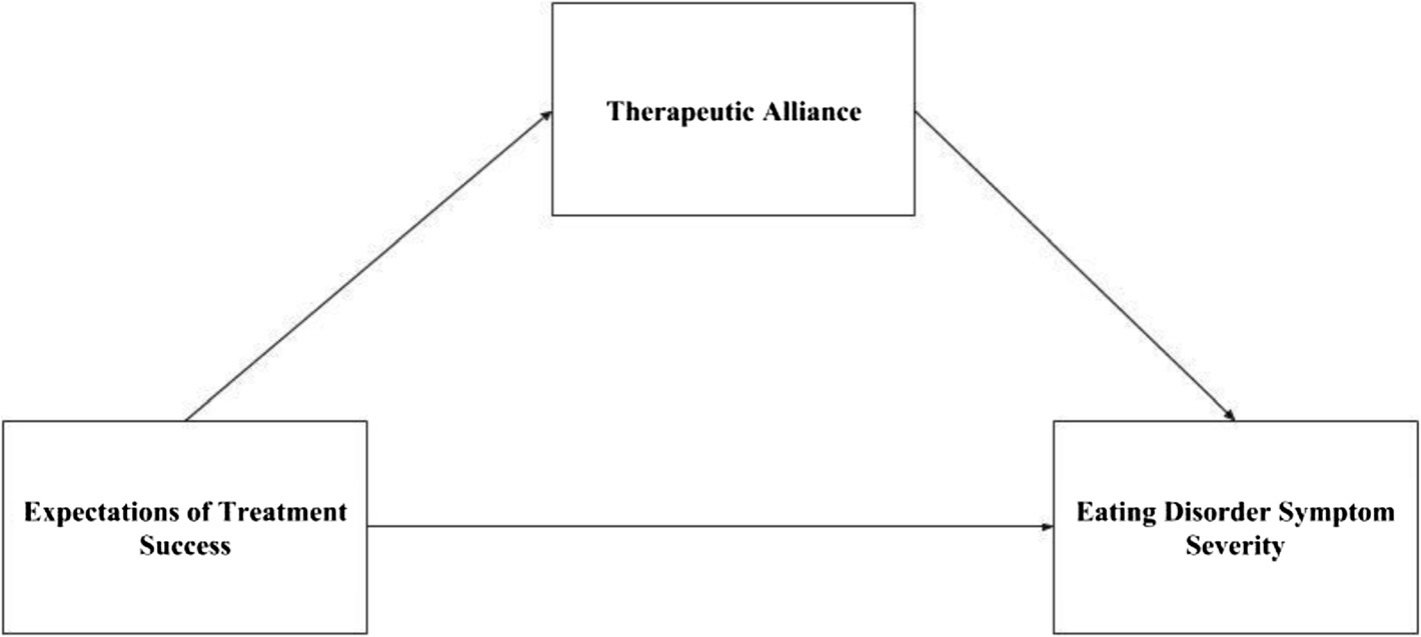
Therapeutic alliance mediates the relationship between treatment expectations and outcome

Given the present findings that anticipated treatment success significantly predicted TA, and past findings from this sample indicating TA as a significant predictor of outcome [19], additional analyses were conducted to determine the possibility of a mediating relationship, such that expectations for therapy impacts overall TA, which impacts treatment outcomes (see Fig. 1). Utilizing Baron and Kenny’s (1986) four step approach, a full mediation effect is suggested for early TA, such that: step 1) expectations of treatment success (TSPE item 2) significantly predicts EOT symptomology (EDE global score; *p* = .005); step 2) expectations of treatment success (TSPE item 2) significantly predicts early TA (*p* < .001); step 3) early TA significantly predicts EOT symptomology (*p* = .02); and step 4) expectations of treatment success no longer significantly predicts EOT symptomology after controlling for early TA (*p* = .07) [36]. A partial mediation effect is suggested for late TA, such that: step 1) expectations of treatment success significantly predicts EOT symptomology (*p* = .005); step 2) expectations of treatment success significantly predicts late TA (*p* < .02); step 3) late TA significantly predicts EOT symptomology (*p* = .002); and step 4) expectations of treatment success significantly predicts EOT symptomology after controlling for early TA (*ps* = .03).

## Discussion

This study examined predictors of TA in a treatment trial offering two psychological therapies for SE-AN. Contrary to our hypotheses, nearly all of the variables examined were not found to be significant predictors of TA at the start of treatment, mid treatment, or EOT. Indeed, patient expectations for treatment, an item in the TSPE which was given following the first therapy session, was found to be the only significant predictor of TA throughout treatment. Mediation analyses suggested that this early treatment factor impacts outcomes such that patient treatment expectations drive the development of TA, which impacts treatment outcomes.

These findings are in contrast to previous research suggesting that increased duration of illness, severity of illness, and decreased motivation may have a negative impact on TA [3, 20–22]. A number of recent studies confirm the difficulties in engaging individuals with SE-AN in psychological treatments [37–39]. This has likely contributed to a prejudice that may be held by many treating clinicians, both in their own expectations for outcome as well as their belief that patients with SE-AN are not interested in positive treatment outcomes. However, it appears that within this sample, illness duration, illness severity, previous failed treatment experiences, and baseline BMI had no significant predictive capacity on patients’ therapeutic relationships. While this study did not include patients with a short duration of AN, reported levels of TA in the current study were consistent with the use of the HRQ with other samples with eating disorders [33]. As such, clinicians should remain highly cautious when assuming that illness characteristics can impact the likelihood of forming an engagement. Additionally, this study found that early TA had little predictive validity on later TA ratings. This suggests that when initial impressions are made, other factors may override them and have a greater influence on the strength of the therapeutic relationship as treatment progresses.

Patients’ belief in the ability of the treatment to contribute to overall improvement of symptoms was the only significant contributor to their experience of TA at all stages of treatment. This finding is consistent with the literature on adults with BN [21], and longstanding work noting the importance of hope and alliance in treatments [40]. Further, it suggests that clinicians attempting to engage patients with SE-AN in meaningful psychological work should focus early in treatment on bolstering patient expectations about the intervention they are undertaking. This may include providing psychoeducation regarding the efficacy of CBT-AN and SSCM for SE-AN [25], or the benefits of symptom change even when remission is not possible [27]. Where available, these early treatment actions may aid the process of building a positive TA throughout treatment, which in turn supports better outcomes [19, 41]. Indeed, as more research is conducted to identify the most effective means to treat adults with SE-AN, bolstering TA is a likely channel to improve outcomes for currently available treatments [42].

To our knowledge, this is the first study to examine predictors of TA in a sample of SE-AN. Engagement in treatments for SE-AN remains a struggle for clinicians. Therefore, examining predictors of TA as a means to enhance the likelihood of forming a positive TA is crucial. There were several strengths to this study, including assessments with well-validated measures. Despite these strengths, limitations should be considered in the interpretation of these findings. While the sample sizes were sufficient to show differences in outcomes, they were also moderate in size. Further, despite its use across a number of RCTs [25, 33–35], the two-item TSPE remains unvalidated. The TSPE was also administered following the first session, which means it is possible that patient expectations were also influenced by the rapport experienced with the therapist during the first session. For this reason, we are cautious to over interpret these findings until they can be confirmed in future research examining treatment expectation prior to meeting the therapist. Additionally, this sample includes only individuals with SE-AN. While it remains unclear how these results generalize to individuals with a shorter duration or less severe presentations, the authors see this as a future research question, rather than a limitation of the study. It is also of note that the number of analyses conducted increases the risk for Type I error. Future studies should explore predictors of TA in larger samples, including both individuals with BN and with patients with a shorter duration of AN. This would also exclude the possibility of any floor effects influencing the non-significant finding for duration of illness arising from the chronic nature of this group. It may also be beneficial to explore patient characteristics in conjunction with therapist characteristics.

## Conclusions

Results of this study suggest that an initial patient expectation for successful outcome significantly predicts patient-reported TA throughout treatment. Further, a mediational relationship between early patient treatment expectations, TA, and outcomes appears to exist for this sample of adults with SE-AN. Most cases of SE-AN are defined for clinicians by patients’ egosyntonic wish to preserve maladaptive behaviors that they identify as having a “functional” purpose [37]. Through this lens, therapeutic relationships can at times be sacrificed, regardless of chronicity. However, recent findings show that positive and strong TA can be established in individuals with SE-AN [19]. The present study expands insight into TA for individuals with SE-AN, demonstrating that TA can be bolstered with early treatment expectations for successful treatment.

## Abbreviations

AN: anorexia nervosa
ANSOCQ: anorexia nervosa stages of change questionnaire
BMI: body mass index
BN: bulimia nervosa
CBT-AN: cognitive behavioral therapy for anorexia nervosa
DSM-IV-TR: Diagnostic and Statistical Manual for Mental Disorders, 4^th^ Edition, Text Revision
EDE: eating disorder examination
EOT: end of treatment
HRQ: helping relationships questionnaire
IRB: Institutional review board
RCT: randomized controlled trial
SE-AN: severe and enduring anorexia nervosa
SSCM: specialist supportive clinical management
TA: therapeutic alliance
TSPE: treatment suitability and patient expectations
